# Patient-reported outcomes after a distal radius fracture in adults: a 3–4 years follow-up

**DOI:** 10.1080/17453674.2019.1568098

**Published:** 2019-01-23

**Authors:** Roderick H van Leerdam, Floortje Huizing, Frank Termaat, Sanne Kleinveld, Steven J Rhemrev, Pieta Krijnen, Inger B Schipper

**Affiliations:** a Department of Surgery, Leiden University Medical Center (LUMC), The Netherlands;; b Department of Surgery, Haga Hospital, The Netherlands;;; c Department of Surgery, The Hague Medical Center (HMC), The Netherlands

## Abstract

Background and purpose — There are few reports on the outcome of distal radius fractures after 1 year. Therefore we investigated the long-term patient-reported functional outcome and health-related quality of life after a distal radius fracture in adults.

Patients and methods — We reviewed 823 patients, treated either nonoperatively or operatively in 2012. After a mean follow-up of 3.8 years 285 patients (35%) completed the Patient-Rated Wrist Evaluation (PRWE) and EuroQol-5D.

Results — The mean PRWE score was 11. The mean EQ-5D index value was 0.88 and the mean EQ VAS for self-rated health status was 80. Nonoperatively treated type A and type B fractures had lower PRWE scores compared with operatively treated patients, whereas the EQ-5D was similar between groups. The EQ VAS for patients aged 65 and older was statistically significantly lower than that of younger patients.

Interpretation — Patients had a good overall long-term functional outcome after a distal radius fracture. Patients with fractures that were possible to treat nonoperatively had less pain and better wrist function after long-term follow-up than patients who needed surgical fixation.

The initial treatment of distal radius fractures can be operative or non-operative. The functional outcome after these fractures may be impaired (Rozental et al. 2002, Chung et al. 2007, Ranjeet and Estrella 2012). Most studies describe the functional outcome over a 1-year period. The recovery is characterized by an initial decline in function followed by improvement after 6–12 months. Though functional recovery is known to often take more than 1 year, studies that describe functional recovery beyond 1 year are scarce and report predominantly on certain subgroups (Brogren et al. 2011, Landgren et al. 2011, Williksen et al. 2013, Lalone et al. 2017).

In recent decades there has been a shift in how to assess the functional outcome after distal radius fractures. Previously, the outcome was predominantly determined by physician-reported parameters such as the radiographic properties of the fracture or range of motion of the injured joint or limb. However, these clinical parameters do not represent patients’ perspectives and seem less relevant for outcome evaluation (Fujii et al. 2002, Anzarut et al. 2004, Wilcke et al. 2007, Finsen et al. 2013, Plant et al. 2017). Nowadays, patient-reported outcomes (PROs) seem to be more relevant and are used as standard for this purpose.

In the initial phase of the treatment, patients are focused on pain relief rather than long-term expectations and consequences. Patient-relevant long-term results include functional recovery, return to work, and the ability to perform daily activities. For distal radius fractures, several PROMs have been developed and validated such as the Patient Rated Wrist Evaluation (PRWE) (Abramo et al. 2008, Kleinlugtenbelt et al. 2016, Landgren et al. 2017, MacDermid 2011) and the Disability of the Arm, Shoulder and Hand score (DASH) (Hudak et al. 1996). To assess a patient’s general health and quality of life a generic PROM like the EuroQol-5D (EQ-5D) or the Short Form 36 (SF-36) can be used (Brooks 1996, Warek and Falkinham 1996). So far, the available validated PROMs for distal radius fractures have been mainly used for research purposes. Although not yet implemented in daily clinical practice, it is assumed that PROMs can be a valuable tool for monitoring outcome during routine clinical follow-up (MacDermid et al. 2004).

Most studies on determining the functional outcomes of distal radius fractures focus on the outcome of PROMs within the first year. We assessed long-term patient-reported outcome after a nonoperative or operatively treated distal radius fracture. 

## Patients and methods

Hospital patient records were reviewed to identify all patients with a distal radius fracture (DRF) that were treated in 1 of the 3 regional Level-1 trauma center locations in the Western part of the Netherlands between January 2012 and December 2012. Yearly, an estimate of 900 distal radius fractures are treated in these centers.

Inclusion criteria were completed treatment for a DRF of any type with a minimum follow-up of 3 years and age ≥ 18 years. Patients were excluded if they could not be followed for at least 3 years (e.g., deceased or living abroad), were mentally incompetent, had more than one (contralateral or ipsilateral) or an open DRF, or had other diseases or injuries that interfered with the normal function of the wrist (e.g., rheumatic diseases or neurodegenerative diseases, multiple injuries). Treatment was either operative or nonoperative. Type of treatment was based on the national treatment guideline for DRFs (Dutch Surgical Society 2010) and supervised by the attending surgeon. At the time of the study (January–April 2016) all included patients had completed their treatment.

Functional outcome was measured using the PRWE. The PRWE is a 15-item questionnaire on wrist pain and disability in activities of daily living as perceived by the patient. Scores on 2 subscales (pain and function) are combined in a total score ranging from 0 (no pain nor disability) to 100 (severe pain and disability) (MacDermid et al. 1998). The total PRWE score was calculated using the published algorithm (sum of the 5 pain items plus the sum of the 10 function items divided by 2) (MacDermid 2011). Questions not answered by patients were replaced with the mean score of the subscale as specified in the PRWE user manual. The patient-perceived health-related quality of life was measured using the EQ-5D questionnaire, a generally accepted and validated PROM (Brooks 1996). The EQ-5D descriptive system has 5 questions regarding mobility, self-care, daily activities, pain/discomfort, and anxiety/depression. The scores for these 5 health dimensions are converted to a utility score that ranges from –0.33 to 1; a lower score reflects a poorer quality of life (Lamers et al. 2005). The EQ Visual Analogue Scale (a vertical 100 mm scale from zero to 100) is a self-rated health status measure.

To identify risk factors for a worse patient-reported functional outcome we recorded age (< 65 years or ≥ 65 years), sex, fracture side, whether the dominant hand was fractured, fracture type according to the AO classification (type A: extra-articular fracture; type B: partial articular fracture; type C: complete articular fracture) and treatment (nonoperative or operative) (Müller et al. 1990, Marsh et al. 2007).

Patients who were eligible according to hospital records received the questionnaires and a written consent form by post in January 2016. To increase the response rate, all non-responders were additionally contacted by phone up to 3 months after the initial questionnaires were sent.

### Statistics

The patient characteristics were described using summary statistics and the response group was compared with the non-response group to study whether the respondents were representative of the total study population. PRWE and EQ-5D scores were compared between patient groups (sex, treatment, dominant hand fractured, AO classification) using t Students’ t-test or 1-way ANOVA. Multiple linear regression analysis was conducted to identify which patient and fracture characteristics (age, sex, fracture type, dominance of the fractured hand, treatment) were associated with the PRWE score. All statistical analyses were performed using SPSS Statistics for Windows, version 23 (IM Corp, Armonk, NY, USA). P-values < 0.05 were considered statistically significant.

### Ethics, funding, and potential conflicts of interest

Prior to the start of the study, the institution’s ethics committee approved the study (P15.311). No financial support for the research was obtained. No conflicts of interest were declared.

## Results

823 patients met the inclusion criteria and were sent the questionnaires by mail. 364 (44%) did not respond and were considered lost to follow-up. Another 174 patients (21%) were unwilling to participate. The response rate was thus 35%. 13 of the 285 responders were excluded: 6 patients suffered from other diseases/injuries that interfered with proper function of the wrist, 5 had more than one DRF, and 2 completed less than 50% of the questionnaire. 207 (73%) responded by mail and 78 by phone, after initially not having responded. 243 (89%) patients filled in the questionnaire completely. Of the incomplete questionnaires no patient left more than 4 of the 33 questions unanswered. Responders and non-responders were similar regarding age, sex, fracture side, and AO fracture classification. The proportion of operatively treated fractures was higher in the responders’ group compared with the non-responders’ group: 32% versus 20% (p < 0.01) (Tables 1 and 2). Mean follow-up was 46 months (42–50).

**Table 1.** Patient characteristics of response and non-response groups

Factor		Response	Non-response	p-value
Number, n (%)		285 (35)	538 (65)	
Mean age (SD) (years)		62 (16)	61 (21)	0.7
Sex, n (%)	Male	71 (25)	157 (29)	0.2
	Female	214 (75)	381 (71)	
AO fracture type, n (%)				
A		113 (40)	250 (47)	0.1
B		90 (32)	161 (30)	
C		82 (29)	127 (24)	
Treatment group, n (%)				
Nonoperative		195 (68)	431 (80)	< 0.001
Operative		90 (32)	107 (20)	
Fractured side, n (%)	Left	159 (56)	286 (53)	0.6
	Right	126 (44)	250 (47)	

**Table 2.** Characteristics of included participants

Patient characteristics		
Number, n		272
Mean age (SD) (years)		62 (16)
Sex, n (%)	Male	69 (25)
	Female	203 (75)
Months of follow-up (SD)		46 (4)
AO fracture type, n (%)	A	107 (39)
	B	86 (32)
	C	79 (29)
Treatment group, n (%)	Non-operative	185 (68)
	Operative	87 (32)
Fractured side, n (%)	Left	149 (55)
Dominant side fractured, n (%)	No	123 (45)
	Missing	53 (20)

### Patient-reported outcome

#### Patient-Rated Wrist Evaluation

The mean PRWE score was 11 (SD 18, range 0–96). There was no statistically significant difference in PRWE scores regarding sex (p = 0.2) or AO fracture type (p = 0.6). Wrist pain and function were similar for patients who fractured their dominant hand (p = 0.3). PRWE scores also did not statistically significantly differ between elderly (65 years and over) and younger patients (p = 0.2). The patients in the nonoperative treatment group had lower PRWE scores, indicating less pain and better wrist function, compared with the patients in the operative treatment group (p < 0.01) (Tables 3 and 4).

**Table 3.** Patient-reported outcomes for PRWE and EQ-5D. Values are mean (SD)

		PRWE	p-value	EQ-5D	p-value	EQ VAS	p-value
Total		11 (18)		0.88 (0.20)		80 (15)	
Age	< 65	10 (16)	0.2	0.90 (0.20)	0.06	82 (15)	0.04
	≥ 65	12 (20)		0.85 (0.20)		78 (16)	
Sex	Male	8 (13)	0.2	0.92 (0.16)	0.09	83 (14)	0.09
	Female	12 (20)		0.87 (0.21)		79 (16)	
AO fracture type							
A		9 (18)	0.6	0.88 (0.20)	0.6	80 (15)	0.9
B		11 (19)		0.89 (0.16)		79 (15)	
C		12 (18)		0.86 (0.23)		80 (16)	
Treatment group							
Nonoperative		8 (15)	< 0.01	0.89 (0.19)	0.4	80 (15)	0.7
Operative		17 (22)		0.86 (0.22)		79 (16)	
Dominant side fractures							
Yes		10 (16)	0.3	0.87 (0.21)	0.9	78 (15)	0.3
No		13 (21)		0.86 (0.22)		80 (16)	

**Table 4.** Mean PRWE score for conservative and operatively treated type A, B, and C fractures. Values are mean (SD)

	Non-operative		Operative treatment		PRWE difference	p-value
	n	PRWE	n	PRWE		
AO fracture type						
A	93	8 (15)	14	20 (29)	–12	0.002
B	67	7 (15)	19	25 (24)	–18	< 0.001
C	25	10 (14)	54	13 (19)	–2.9	0.2

In the multiple linear regression analysis the nonoperatively treated patients with a type A fracture scored better than operatively treated type A fractures; this was borderline significant (p = 0.06). The PRWE score for type B fractures was favorable in the nonoperative group (p < 0.01). No significant difference between PRWE score of non-operatively or operatively treated C fractures was noted (Table 5, see Supplementary data).

**Table 5.** Multiple linear regression analysis of PRWE scores per fracture type

	n	Regression coefficient	(95% CI)	p-value
**Type A fractures**				
Age	107	0.28	(–0.03 to 0.59)	0.08
Sex				
Male	20	(ref)		
Female	87	–5.4	(–17 to 6.6)	0.4
Dominant side fracture				
Yes	36	(ref)		
No	45	3.8	(–4.9 to 12)	0.4
Treatment				
Non-operative	93	(ref)		
Operative	14	11	(–0.5 to 23)	0.06
**Type B fractures**				
Age	86	0.08	(–0.20 to 0.36)	0.6
Sex				
Male	27	(ref)		
Female	59	7.9	(–1.6 to 17)	0.1
Dominant side fracture				
Yes	32	(ref)		
No	41	2.9	(–5.6 to 11)	0.5
Treatment				
Non-operative	67	(ref)		
Operative	19	16	(5.6 to 25)	< 0.01
**Type C fractures**				
Age	79	–0.32	(–0.66 to 0.02)	0.07
Sex				
Male	22	(ref)		
Female	57	11	(–0.36 to 22)	0.06
Dominant side fracture				
Yes	28	(ref)		
No	37	–0.78	(–10 to 8)	0.9
Treatment				
Non-operative	25	(ref)		
Operative	54	2.0	(–7.6 to 12)	0.7

Ref = reference group.

#### EuroQoL-5D

The mean EQ-5D score after follow-up was 0.88. EQ-5D scores were similar between AO fracture type groups or between the treatment groups. The mean EQ-5D score for patients aged 65 and older was lower than that of younger patients (p = 0.06).

The mean EQ VAS was 80 (range 30–100; 95% CI 78–82). The EQ VAS score for patients aged 65 and older was lower than that of younger patients (p = 0.04). There were no significant differences in the EQ VAS between other subgroups (Table 3). 

## Discussion

The patients in this study, with a mean age of 62 years, had a good overall long-term functional outcome after a DRF. Nonoperatively treated type A and B fractures had better PRWE scores than operatively treated type A and B fractures after a mean follow-up of almost 4 years indicating better wrist function with less pain. In multiple linear regression analysis this difference was only statistically significant for type B fractures. We found similar patient-reported functional outcome between nonoperatively treated type A, B, and C fractures. Higher scores for health-related quality of life as measured by the EQ-5D did not go hand in hand with better wrist function, as measured by PRWE.

Most previous studies on patient-reported functional outcome presented results obtained after 3, 6, or 12 months of follow-up. Our study assessed patient-reported outcomes at a mean of 4 years. We found a significant difference in functional outcome between the 2 treatment groups, in favor of nonoperative treatment. In the subgroup analysis this functional benefit was most obvious in type A and B fractures. The minimum clinically relevant difference of the PRWE score for patients with a DRF has been reported to vary from 4 to 20 (Kim and Kang 2013, Walenkamp et al. 2015, Kleinlugtenbelt et al. 2018). We found a mean difference of 12 and 18 between operatively and nonoperatively treated type A and B fractures respectively, which could indicate a clinically relevant difference.

A number of papers have compared patient-reported functional outcome between nonoperative and operative treatment in elderly patients. Arora et al. (2011) found significantly better patient-reported wrist function for type A and C fractures in the operative treatment group in the early postoperative time period, yet after 6 and 12 months only grip strength was statistically significantly better. Egol et al. (2010) did not report the functional benefit for operatively treated patients. At 3, 6, and 12 months there was no significant difference in patient-reported wrist function. Research by Bartl et al. (2014) concerning type C fractures only found a marginal and clinically unimportant difference in functionality in favor of operatively treated patients at 3 and 12 months’ follow-up. Similar to our results, quality of life was similar between operative and nonoperative treatment. In contrast with these 3 studies and our findings, Sharma et al. (2014) found statistically significantly better wrist function in operatively treated type B and C fractures at the final follow-up, after 24 months. To our knowledge no other studies have reported on the long-term follow-up patient-reported functional outcome in favor of nonoperative treatment.

In our study the EQ-5D showed no statistically significant differences in outcome for age, sex, treatment group, or fracture type. An earlier study implied that the EQ-5D values might normalize to pre-fracture values at 6–12 months (Hagino et al. 2009). Because of these contradictory results we feel that the EQ-5D may not be the most discriminating and appropriate instrument in evaluation of long-term follow-up in DRFs.

One of the strengths of this study was the large number of patients who participated. This provided considerable statistical power to the analysis (Table 2). Our population included all types of and differently treated distal radius fractures. In the subgroup analysis, however, the group sizes were relatively small and hence statistical analysis was not meaningful. Another limitation is a greater proportion of operatively treated patients in the response group, which introduces a potential bias. The over-representation may indicate that the operated patients had worse results compared with the nonoperatively treated patients. The willingness to respond to the questionnaire might be influenced by the residual level of pain and dysfunction; operatively treated patients might be more eager to share their experiences. Another explanation could be that having surgery is a big life event, triggering a higher rate of response. Also, more type C fractures were present in the operated group. This is merely due to the national guideline, which advises that complex fractures be treated more often with internal or external fixation. However, we did not find statistically significant differences in functional outcome between fracture types, indicating that in our study function did not correlate with fracture type.

Another shortcoming was the low response rate of 35% due to a high number of non-responding or unwilling-to-participate patients. To increase the response rate, all non-responders were additionally contacted by phone up to 3 months after the initial questionnaires were sent. This introduces a risk of bias. Finally, the type of surgery and experience of the surgeon was not investigated. Both parameters could also affect outcome (Landgren et al. 2017). They were, however, beyond the scope of our study.

Operative treatment for type A and B wrist fractures was associated with worse patient-reported wrist function at prolonged follow-up compared with nonoperative treatment. We speculate that this is possibly due to a higher complication rate after operative treatment, such as symptomatic hardware and hardware-related tendon irritation or even injury that can occur up to 4 years after surgery (Soong et al. 2011). Most of the published studies evaluate complications up to one year after operation. We did not, though, have the data in this study to substantiate this speculation. However, further analysis of the records of operatively treated patients showed that, in 9 out of the 87 patients, hardware was removed because of functional limitations and pain. This could even be an underestimation if patients sought care elsewhere. The mean PRWE score of patients in this study who had their hardware removed was 24 compared with 16 in patients who did not. Although this group was too small to perform statistical analysis, it should be noted that after reoperation patient wrist function was still not satisfactory.

An alternative explanation for the difference between the 2 treatment groups could be that patients who were treated operatively have different expectations than patients who were treated nonoperatively. The treating surgeon must be aware of this: expectation management plays a crucial role in satisfaction with final functional outcome.

In summary, after a mean follow-up of 4 years, patients generally perceived the outcome after a distal radius fracture as good. Patients with fractures that it was deemed possible to treat nonoperatively had better PRWE scores compared with those needing operative treatment independent of sex and affected side. Quality of life between the treatment groups was similar.

### Supplementary data

Table 5 is available as supplementary data in the online version of this article, http://dx.doi.org/ 10.1080/17453674. 2019.1568098

## Supplementary Material

Supplemental Material
