# Supplementary material for: Patient-reported outcomes after a distal radius fracture in adults: a 3–4 years follow-up
Source: Acta Orthop. 2019 Jan 23;90(2):129–34. doi: 10.1080/17453674.2019.1568098 (PMC6461106; doi:10.1080/17453674.2019.1568098)
Supplement: Supplemental Material [file IORT_A_1568098_SM5510.pdf]

Supplementary data

Table 5. Multiple linear regression analysis of PRWE scores per fracture type

	n	Regression coefficient	(95% CI)	p-value
**Type A fractures**				
Age	107	0.28	(–0.03 to 0.59)	0.08
Sex				
Male	20	(ref)		
Female	87	–5.4	(–17 to 6.6)	0.4
Dominant side fracture				
Yes	36	(ref)		
No	45	3.8	(–4.9 to 12)	0.4
Treatment				
Non-operative	93	(ref)		
Operative	14	11	(–0.5 to 23)	0.06
**Type B fractures**				
Age	86	0.08	(–0.20 to 0.36)	0.6
Sex				
Male	27	(ref)		
Female	59	7.9	(–1.6 to 17)	0.1
Dominant side fracture				
Yes	32	(ref)		
No	41	2.9	(–5.6 to 11)	0.5
Treatment				
Non-operative	67	(ref)		
Operative	19	16	(5.6 to 25)	< 0.01
**Type C fractures**				
Age	79	–0.32	(–0.66 to 0.02)	0.07
Sex				
Male	22	(ref)		
Female	57	11	(–0.36 to 22)	0.06
Dominant side fracture				
Yes	28	(ref)		
No	37	–0.78	(–10 to 8)	0.9
Treatment				
Non-operative	25	(ref)		
Operative	54	2.0	(–7.6 to 12)	0.7

Ref = reference group.
